# Pupillary Response to Negative Emotional Stimuli Is Differentially Affected in Meditation Practitioners

**DOI:** 10.3389/fnhum.2017.00209

**Published:** 2017-05-03

**Authors:** Alejandra Vasquez-Rosati, Enzo P. Brunetti, Carmen Cordero, Pedro E. Maldonado

**Affiliations:** ^1^Biomedical Neuroscience Institute, Faculty of Medicine, Universidad de ChileSantiago, Chile; ^2^Institute of Biomedical Sciences (ICBM), Faculty of Medicine, Universidad de ChileSantiago, Chile; ^3^Centro de Integración Cognitivo CorporalSantiago, Chile; ^4^Department of Industrial Engineering (DII), Universidad de ChileSantiago, Chile

**Keywords:** autonomic nervous system, pupil size, mindfulness meditation, images, emotion

## Abstract

Clinically, meditative practices have become increasingly relevant, decreasing anxiety in patients and increasing antibody production. However, few studies have examined the physiological correlates, or effects of the incorporation of meditative practices. Because pupillary reactivity is a marker for autonomic changes and emotional processing, we hypothesized that the pupillary responses of mindfulness meditation practitioners (MP) and subjects without such practices (non-meditators (NM)) differ, reflecting different emotional processing. In a group of 11 MP and 9 NM, we recorded the pupil diameter using video-oculography while subjects explored images with emotional contents. Although both groups showed a similar pupillary response for positive and neutral images, negative images evoked a greater pupillary contraction and a weaker dilation in the MP group. Also, this group had faster physiological recovery to baseline levels. These results suggest that mindfulness meditation practices modulate the response of the autonomic nervous system, reflected in the pupillary response to negative images and faster physiological recovery to baseline levels, suggesting that pupillometry could be used to assess the potential health benefits of these practices in patients.

## Introduction

The term “meditation” refers to a wide variety of attentional practices. Lutz et al. (2008b) conceptualized meditation as a complex family of attentional regulatory strategies developed for various purposes, including cultivating emotional well-being and balance. There are multiple benefits associated with these practices that include behavioral, attentional and physiological changes. Among the reported benefits are decreased level of anxiety (Chiesa and Serretti, 2010), decrease of primary symptoms in children diagnosed with attentional deficit hyperactivity disorder (Harrison, 2004; Rubia, 2009), positive effects on attention enhancing the functioning of specific subcomponents of attention (Jha et al., 2007; Tang et al., 2009), decrease in the thinning of some areas of the cerebral cortex (Lazar et al., 2006), improving immune function (Davidson et al., 2003) and maintaining telomerase activity and immune cell longevity (Jacobs et al., 2011). Nonetheless, a key aspect of the practice of meditation relates to their impact on affective responses. However, little is known about the neuronal mechanisms involved in meditation practitioners (MP), and there have been few experimental approaches on the physiological and autonomic effects of the incorporation of these practices (Lutz et al., 2008a,b, 2009).

Looking for the neuronal substrates that participate in meditation, Lutz et al. (2008a) showed how expert meditators practitioners, in compassion meditation, have different BOLD responses in the insular cortex when they listen to sounds with emotional valence in relation with novice subjects. In this practice, meditators focus on particular persons or groups of beings reaching a state of “pure compassion” or “non-referential compassion.” Results show that in all subjects the BOLD signal in the anterior insular cortex (AI) and anterior cingular cortex (ACC) was greater to all emotional sounds during compassion meditation compared with rest. Also, expert meditators showed stronger responses in somatosensorial regions for negative sounds compared with positive and neutral sounds and compared with novice subjects. The activity of the insular cortex has been physiologically linked to some autonomic markers. Lutz et al. (2009) correlated changes in the blood flow in the insular cortex with higher heart rate variability (HRV), demonstrating a relation with behaviorally observed physiological changes.

The pupil diameter has also been explored as a physiological marker of the autonomic response associated with auditory (Partala and Surakka, 2003) and visual (Bradley et al., 2008) emotional processing. Bradley et al. (2008) presented to normal subjects a series of images with different emotional valence (pleasant, unpleasant and neutral), and observed that the pupillary responses were greater for pleasant and unpleasant images compared with the neutral ones, confirming that pupillary changes not only respond to luminance but also respond to hedonic valence. Later, Onorati et al. (2013) showed that the dynamics of the pupil have similar autonomic fluctuations than both high and low HRV frequency bands, validating the pupil dynamics as a marker of psychophysiological changes related to simple affective states.

Considering that meditators practitioners have different physiological responses to emotional stimuli, which include the autonomic system, would pupil response reflect this different emotional processing in mindfulness MP? We hypothesized that the pupil, as an effector of the autonomic responses associated with the differential emotional processing, would manifest in the diameter different fluctuations in mindfulness MP compared with non-practitioners, associated to the exposure to natural images with emotional valence. Moreover, because this kind of attentional practice is similar to focus attention mindfulness meditation and centered in proprioception, we expected distinct reactivity to emotional images in this class of MP.

To validate this hypothesis, we studied the pupillary responses to a collection of natural images with standardized emotional valence between a group of mindfulness MP and matching controls. To maintain homogeneity, we recruited mediators from a single meditation center whose practice is centered in proprioceptive consciousness and consisted in focusing and maintaining the attention on their breathing, directing the focus to different parts of the body, and then making specific and controlled body movements while aware monitoring the body and breathing. We aimed to determine whether changes in pupil diameter would serve as a reliable marker of the emotional processing in meditators, and as a predictor of the influence of this meditation practice. Because pupillary response has an exceptionally high-resolution time course compared to other autonomic measures, it would also allow correlating the autonomic changes during emotional processing with other variables, as neuronal activity, thus contributing to the understanding of beneficial physiological changes involved in these behaviors.

## Materials and Methods

### Participants

We recruited 14 subjects that practice meditation (MP) and 10 control subjects that declared to have no meditation training (non-meditators (NM)). This study was carried out in accordance with the recommendations of the University ethics committee (Comité de Ética de Investigación en Seres Humanos from the Facultad de Medicina, Universidad de Chile, Santiago, Chile), with written informed consent from all subjects. All subjects gave written informed consent in accordance with the Declaration of Helsinki. The protocol and consent form was approved by the Comité de Ética de Investigación en Seres Humanos. Two MP subjects were excluded from the study because they interrupted their practices for a year or more, and one subject was excluded because muscle movements generated extremely poor signals. One NM was excluded because he did practice other techniques of emotional regulation. Our final sample included 11 MP and 9 NM subjects. All subjects were college graduates. Average ages for MP and NM groups were 44.6 ± 10.27 and 39 ± 12.8 years, respectively. Nine out of 11 MP were women, compared to 8 out of 9 in the NM group. Body mass index yielded 23.07 ± 3.2 for MP and 23.16 ± 4.3 for NM subjects. All subjects presented a normal or corrected-normal vision and had not consumed medication that either affects the autonomic neural system or the pupillary responses (cholinergic, opiates, phenothiazine, sedative-hypnotics, antihistamines, antidepressants, atropine). The training of the experimental subjects consisted in a weekly practice (15 min daily and 1 h twice in a week) at a local mediation center (Centro de Integración Cognitivo Corporal, Santiago, Chile), with training experience ranging between 2 and 15 years. All subjects were trained on the same type of meditation, consisting in focusing and maintaining attention on their breathing, then directing the focus to other regions of the body, and making specific and controlled movements, monitoring the corporal sensations, always maintaining focus in their breathing cycle.

### Task and Measurements

**Figure 1:**
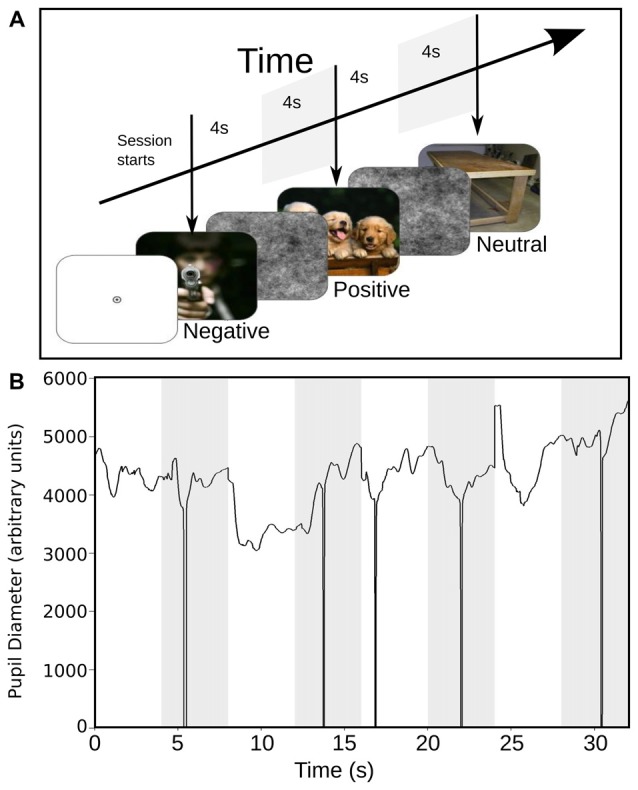
**(A)** Stimulus scheme and timing sequence. Each trial started with the presentation of a full-color image with emotional content during 4 s (the images presented in this figure are not actual samples from the International Affective Picture System (IAPS) collection). This image was followed by the presentation of a black and white image of pink noise, for another 4 s. The images with different emotional content were presented in a random sequence. **(B)** Raw data with pupillary size, in response to the presentation of a sequence of images. The white epoch starting at time 0 represents the time course of pupil size during the response to emotional images, and the shaded area represents the corresponding pupil size to pink-noise images. The downward spikes depict blinks that were removed by linear interpolation.

Commercial software (Experiment Builder, SR-Research) controlled all aspects of task presentation. The experimental paradigm consisted of the sequential presentation of 180 color images, selected from the International Affective Picture System (IAPS) collection (Lang et al., 2008). Images were equally divided between positive, negative or neutral categories of emotional valences (60 images each group). The luminance of each image was measured using a commercial photometer (LX-101, Lutron Electronics). The positive, negative, neutral and pink-noise images have a mean luminance (cd/m^2^) of 15.23 (±6.34), 21.23 (±13.32), 19.23 (±8.53) and 14.69 (±3.98) respectively. Each trial was defined as the consecutive presentation of an emotional image for 4 s replaced immediately by a gray-scale pink-noise image presented for 3–4 s. Emotional images were presented, in a pseudo-random but identical order across subjects. Images with the same type of emotional content were interleaved, and no more than three consecutive times, images of the same valence were allowed to be presented. Pink-noise images, which served to lead the pupillary diameter to a steady-state and to eliminate the previous-trial effect (Figure 1A). Sequences were divided into six blocks of 30 trials, with a 2-min rest period. The images were presented via a ViewSonic P815 de 21″ monitor placed at 57 cm of the subject’s eyes. Images spatial dimensions were 40° horizontal and 30° vertical. The subjects rest their chin in a plastic piece that they accommodate to their complete comfort. The subjects also were instructed to keep their attention in the breathing sensation during the task irrespective of their experimental group, while freely viewing the natural images. Subjects were sitting in a comfortable chair, in a dim lit, sound-attenuated and electromagnetically shielded room. Eye monitoring and pupil size measurements were recorded in arbitrary units at 500 Hz and 16 bits of precision, using a video-oculagraphic head-positioned commercial system (EyeLink 2, SR Research Ltd., Kanata, ON, Canada). Also, we continuously measured the basal heart rate (HR) of all participants using a custom-made photoplethysmograph, to contrast our pupillary measurement with another autonomic variable. After the recording session, participants evaluated the subjective perception of each image using the self-assessment manikin (SAM, Bradley and Lang, 1994; Lang et al., 2008), a graphic figure nine-point scale. The valence dimension ranges from a frowning unhappy figure to a smiling happy figure. Meanwhile, the arousal dimension ranges from a relaxed, sleepy figure to an exited wide-eyed figure. Each image was presented again for 4 s, followed by a 4-s interval in which the subject used the paper-and-pencil version of SAM to rate experienced pleasure and arousal while viewing the picture.

### Data Analysis

All data analysis was done using Matlab (The Mathworks, Inc., Natick, MA, USA). Data visualization, artifacts detection/correction and elimination of single epochs were done using a custom graphical-user-interface. Epochs spanned the period between 200 ms previous to the onset of the emotional image, and 8000 ms after that period, which included the pink-noise image presentation. First, we eliminated epochs containing eye-blinks in the interval between 200 ms before and after the onset of the stimuli and around the maximal pupil contraction. The percentage of rejected trials for NM was 20.43% (±11%) and for MP 35.94% (±18.62%). Blink artifacts occurring later in the trials were automatically detected and removed by linear interpolation. Signal offsets due to saccades were detected by thresholding the acceleration signal of the pupillary response and removed by realigning upward or downward the signal. After this process, a baseline correction was applied by subtracting for the whole signal of each trial, the average signal of the 100 ms epoch before stimulus onset. The artifacts-free signals were grouped for each category and averaged for each subject. Because the resting pupil size and pupil reactivity amplitudes may vary across different individuals, we normalized the average signals obtained for each category and subject before averaging across subjects. Normalization was computed by dividing mean signals of all categories by the absolute value of the maximal pupil contraction obtained for the neutral condition, in each subject. This procedure guarantees that maximal pupil contraction for the neutral condition (negative for all subjects) had a value of −1 for each subject while maintaining the magnitude relations between conditions, across subjects. While neutral condition had an equivalent value of maximal pupil contraction for all subjects, this did not necessarily result in an average neutral condition with the same value across subjects, because of the temporal jitter observed in the maximal pupil contraction between all subjects. In order to compare pupillary signals of each category between mediator practitioners (MP) and NM, we measured several parameters of the pupillary responses such as maximal contraction (minimum magnitude between 200 ms and 1500 ms), maximal dilation (maximum magnitude between 800 ms and 2400 ms), speed of contraction and dilation (first derivative of contraction and dilatation curves, respectively), and latency of the maximal contraction. To evaluate the significance of differences among groups we used non-parametric statistical analysis (Kruskal-Wallis and Wilcoxon Rank Sum test).

Finally, in order to compare directly the influence of meditation on pupillary contraction and the capacity of our data, to predict between meditators and NM, we perform a logistic regression analysis, where the dependent variable is categorical and the predictor variable is the maximal pupillary contraction.

## Results

### SAM Ratings

Table 1 shows SAM ratings of both groups. Significant differences were found in valence and arousal ratings between groups. Regarding valence, the MP group rated as more positive of all group of images than the NM group. Also, the NM group rated as more activating negative images than the MP. These results suggest that the MP group have an attenuated subjective perception of negative images compared to the NM group. These differences validated differential perception of the emotional image between both groups and prompted us to examine further differences in the autonomic responses. Further, we sought for correlations between pupil changes and perceived emotion of the images but found no significant correlations.

**Table 1:** **International Affective Picture System (IAPS) subjective evaluation by self-assessment manikin (SAM) scale**.

	Non Meditators			Meditators		
	Positive	Negative	Neutral	Positive	Negative	Neutral
Valence	6.32**	1.61**	4.45**	7.20**	2.53**	5.25**
Activation	3.39	7.18**	3.41	3.36	6.12**	3.85

**p < 0.00 between group differences.

### Pupillary Reactivity to Emotional Images

**Figure 2:**
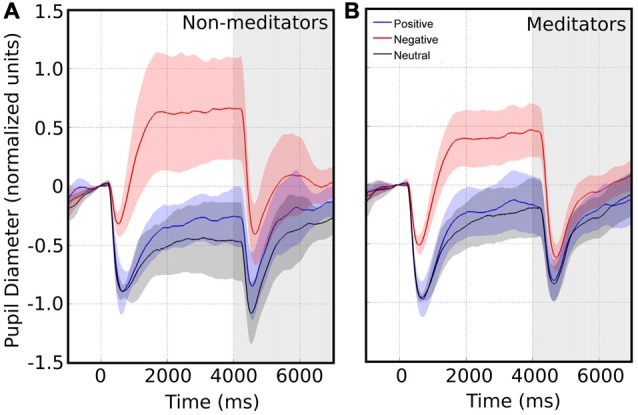
**Average pupillary size response in response to images with emotional valence**. The white area represents the pupillary response when presented with emotional valence image. Time 0 indicates the time of the emotional image stimulus presentation. The shaded area represents the pupil changes in response to pink noise. Confidence intervals for each emotional valence are depicted by the corresponding color shadow. Left and right panels shows the pupillary response for non-meditators (NM) **(A)** and meditationpractitioners (MP) **(B)** respectively.

We collected pupillary traces in a total of 20 subjects. An example raw data recording for four single epochs is shown in Figure 1B. We observed that the pupil size started to change after 200 ms following image presentation. Typically, the pupil shows a fast contraction, followed by a slower dilatation. Figure 2A shows the average pupil signals obtained for each condition for the NM group. For neutral images, maximal pupil contraction peaked at 810 ms from the stimulus onset. This initial contraction was followed by a dilatation that did not reach baseline level by the end of the image presentation. This suggests that these images can establish a different pupillary tone as reflected by the prolongation of the under-baseline pupil response. Later, when the natural image was replaced by the pink-noise image, another contraction took place, and despite its greater magnitude, the pupil size returned rapidly to baseline levels. The difference between the steady state reached by the pupillary response after 2 s in the natural images and the pink-noise images cannot be explained by luminance alone since the luminance of the images were comparable among these categories. Indeed, we found no significant difference in luminance between these images (*F*_(2,87)_ = 2.89, *p* = 0.06). In the same experimental group, the pupillary response associated to the positive category displayed a similar contraction and dilation profile. As before, dilation reached a steady state level below the baseline. However, this steady state (between 2 s and 4 s) reached a greater magnitude at peak compared to neutral images. We calculated windowed statistics to detect latency differences, but we found a continuous overlap in these intervals (see shaded areas in Figure 2A). As with neutral images, pink-noise images after positive images resulted in a pupillary contraction followed by a dilatation with a rapid tendency to return to baseline.

On the other hand, pupillary responses to negative images exhibited a distinctive temporal profile. First, contraction occurred significantly faster, peaking at an earlier latency -more than 100 ms earlier-, and reached a smaller magnitude compared to the other conditions (Kruskal-Wallis, *p* = 0.039). This contraction was followed by a dilatation that quickly exceeded baseline, reaching an over-baseline steady state that remained as long as the time that image was present. This response suggests the participation of an active process in pupil modulation, recruited differently from those occurring for the other two types of images. For pink-noise images presented after negative images, we observed a large contraction, followed by a continuous dilatation that again rapidly tended to baseline values. Table 2 summarizes all measurements computed for several pupillary parameters. Overall, the pupillary changes to negative images exhibited smaller, slower contraction, followed for larger and faster dilatation.

**Table 2:** **Average pupillary parameters and statistical differences within and between groups**.

	Non Meditators			Meditators		
	Positive	Negative	Neutral	Positive	Negative	Neutral
Maximal contraction (nu)	−0.9577	−0.3608^b/c^	−1	−1.0113	−0.5325^b/c^	−1
Maximal dilation (nu)	−0.2913^a^	0.6726^b^	−0.4226	−0.1909	0.4447^b^	−0.2725
Latency of contraction (ms)	794.66	552.88^b^	810.66	685.45	594.5^b^	690.54
Contraction velocity (nu/ms)	−0.0078	−0.0043^b^	−0.0086	−0.0083	−0.0055^b^	−0.0086
Dilation velocity (nu/ms)	0.0018	0.0029^b^	0.0020	0.0024^b^	0.0027^b^	0.0021

*^a^p < 0.05; ^b^p < 0.01 (significant differences against neutral images); p < 0.05 ^c^(significant differences between MP and NM groups)*.

**Figure 3:**
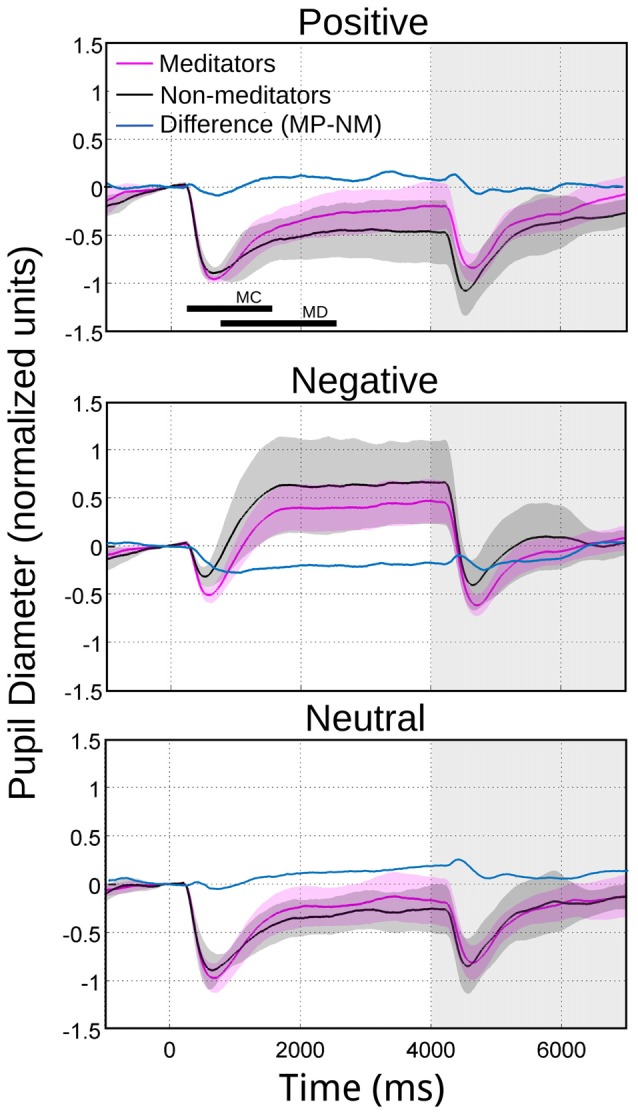
**Average pupillary reactivity in response to images with emotional valence**. NM in black. MP in purple. In blue we indicated the algebraical difference. The bars in the first panel indicate the intervals considered for maximal contraction MC (minimum magnitude between 200 ms and 1500 ms) and maximal dilation MD (maximum magnitude between 800 ms and 2400 ms). During the maximum pupillary contraction for the presentation of negative images (middle panel), the shaded color area does not overlap, indicating significant differences between NM and MP.

The meditators group MP (Figure 2B), on the other hand, shows a similar time course and magnitude changes to the control group for neutral and positive images, but with a significant faster dilatation velocity for negative condition (Kruskal-Wallis negative vs. positive, *p* = 0.002; Kruskal-Wallis negative vs. neutral, *p* = 0.001). Also, as a control group, MP pupillary responses to negative images exhibited smaller, slower contraction, followed for larger and faster dilatation. Figure 3 shows independent comparisons of pupillary responses between groups across all categories. Pupil size curves for neutral and positive images were very similar between groups. For the negative category, we observed in the MP group a larger and more prolonged contraction (Wilcoxon test, *p* = 0.0276), followed by a smaller steady state dilatation (not significant). Contraction velocity tended to be faster in the same group, not reaching statistical significance (Wilcoxon test, *p* = 0.06). Overall, these results show that MP responded differently than NM particularly to images with negative emotional content, which involves the contraction and dilation dynamics.

**Figure 4:**
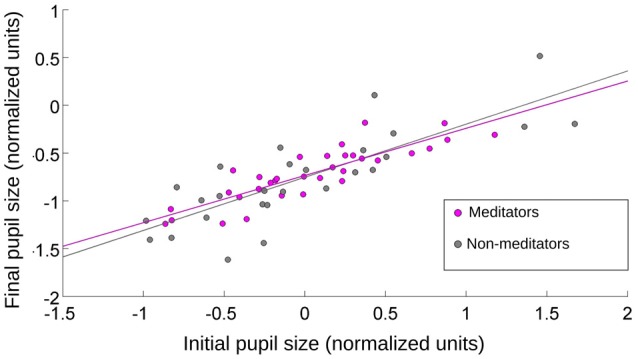
**The relationship between initial and final pupil size after the presentation of pink-noise images**. Initial value was taken at the onset of the image presentation, and final value was pupil size at maximum contraction. For MP and NM, the pupillary contraction was proportional to the initial size of the pupil. *r* = 0.8 (Spearman, *p* = 0.0009).

How much of the initial pupil contraction was explained by the stimulus onset? The average luminance between the emotional and pink-noise images was not significantly different. If the initial pupil contraction was mainly a consequence of a change in the different image’s luminance levels, the magnitude of the contraction should be correlated with the pupil size immediately before the new image presentation. If pupil contraction served only to control the amount of input light arriving at the retina, this contraction must reach a comparable magnitude for the same luminance levels, irrespective of the preceding pupil size. This would translate in greater contractions for greater preceding pupil sizes. We found no correlation between the value of pupil size before the emotional images presentation and the maximum pupil contraction. However, we did find a correlation of *r* = 0.8 (Spearman, *p* = 0.0003) between the initial pupil size (4000 ms) and the maximal contraction of the pupil for the pink noise, for both NM and MP groups (Figure 4). This shows that pupillary contraction generated by the pink-noise images were proportional to the initial size of the pupil, suggesting that when these images were presented the pupil size was mainly driven by the corresponding level of luminance of these images. For images with emotional content, pupil size changes were not explained by luminance, showing that even for the initial pupillary contraction phase there was a strong modulation of both sympathetic and parasympathetic autonomic components.

**Figure 5:**
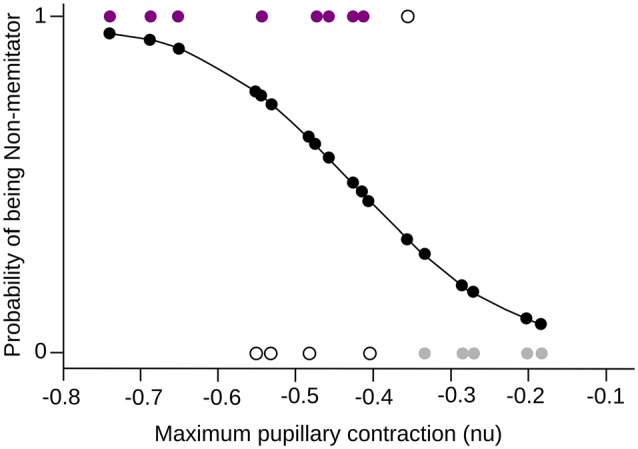
**Logistic Regression**. The maximum pupillary contraction value and the probability of being a mindfulness meditator are shown in the *x*-axis and *y-axis* respectively. The probability curve of being a meditator is shown in black, where 0 is NM and 1 MP. The colored dots (purple for MP, gray for NM) are the subjects that were correctly classified by the model, while the white dots are those subjects that did not fit into the corresponding group.

In order to formalize the relationship between meditation and pupil reactivity, we performed a logistic regression. The classification model obtained using this logistic regression was found to be significant when the maximal contraction to negative images was used (*χ*^2^ = 7.02, *df* = 1, *p* = 0.008, Pseudo *R*^2^ = 0.396). Specifically, we observed that the maximal pupil contraction presented a significant negative coefficient (*β* = −9.84, *Z* = −2.08, *p* = 0.03), indicating that as the maximal contraction increase, the likelihood of being a meditator is reduced. This model present an accuracy of classification using a likelihood of 0.45 as a cutoff of 75% (Figure 5).

### Pupillary Reactivity to Pink-Noise Images

Another finding related to pupillary dynamics was the recovery of the pupil to baseline levels. As mentioned before, this happened after the presentation of pink-noise images. Interestingly, MP pupil restitution is faster than in NM. Figure 2A (in gray background) show that NM followed the pupillary constriction at second 6, and 7 are significant differences between positive and neutral (second 6 *p* = 0.007; second 7 *p* = 0.02), and negative and neutral conditions (second 6 *p* = 0.004; second 7 *p* = 0.04), meanwhile at second 8, between positive emotional conditions against neutral (*p* = 0.04; Table 3). In MP group (Figure 2B), these differences were only for the negative condition (second 6 *p* = 0.02 and second 7 *p* = 0.02) and disappeared at second 8 (Table 3). These results suggest that MP have a faster physiological recovery when are exposed to emotional stimuli than NM.

**Table 3:** **Average pupillary dilation (nu) and statistical differences within groups for pink-noise images**.

	Non Meditators			Meditators		
Second	Positive	Negative	Neutral	Positive	Negative	Neutral
6	−0.191**	0.090**	−0.3618	−0.201	−0.056**	−0.278
7	−0.126*	0.032	−0.269	−0.123	−0.082	−0.075
8	−0.205	−0.118*	−0.205	−0178	−0.206	−0.163

**p < 0.01 within group differences against neutral images; *p < 0.05 within group differences against neutral images.

**Figure 6:**
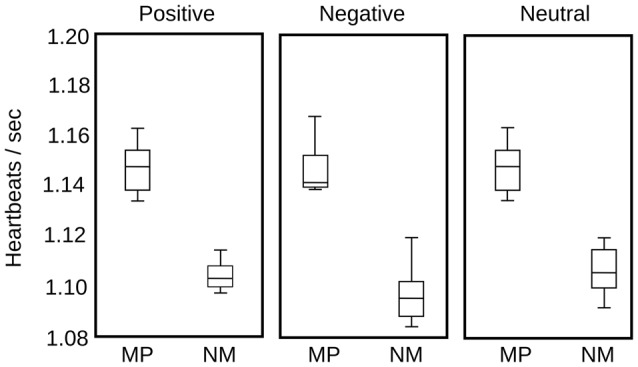
**Heart rate (HR) comparison for MP and NM for the three categories of emotional images**. In each box, the central mark represents the median, the edges of the box are the 25th and 75th percentiles, respectively. The brackets extend to the most extreme data points in the sample. Significant differences were found only between NM and MP groups (*p* = 0.009), but not between image valences.

Finally, in parallel with pupil diameter, we recorded HR in eight MP and eight NM subjects, to examine the putative modifications in other physiological variables, as a consequence of the modulation of the autonomic nervous system. Compared to the time course of pupillary changes, HR modulation is highly variable among individuals. Here, we computed instantaneous frequencies, measuring each time interval and assigning this time to a time bin between the two events. For our large set of stimulus presentations, this approach yielded a large data set of instantaneous frequencies distributed in the time epoch used to explore pupillary change. We then grouped the average HR in 1 s bins and plotted the distribution statistics for each type of emotional images. As is apparent in Figure 6, we found no significant modulation of the HR as a consequence of the presentation of different emotional images. The largest difference was observed in the MP group between negative and neutral or positive images (*p* = 0.054). However, we found that the basal HR of the MP group was significantly lower than those of the NM (*p* = 0.009). These suggest that while we show no immediate changes in HR during image presentations, the practice of mediation appears to have an autonomic tone manifested in a slower HR that was constant during our test.

## Discussion

The results presented here show that images with negative emotional contents trigger distinct pupillary reactivity. While neutral and positive images are mostly characterized by a fast contraction followed by a sustained dilatation under baseline, negative images trigger a slower and smaller contraction, followed by a large, sustained dilatation that maintains during image exploration. These dynamical changes of the pupil size were observed for both meditators and non-practitioners groups. However, a significant difference observed between the two groups is that meditators show an overall diminished sympathetic (noradrenergic) response to negative images. This was manifested mostly as a stronger pupil contraction followed by a weaker dilatation. Our results showed that in meditators, pupillary response to negative images is closer to that of neutral images than in non-practitioners.

A few studies have already examined pupillary changes as a marker for autonomic modulation of emotional images processing (Bradley et al., 2008; Franzen et al., 2009; Dietz et al., 2011). These studies differ between them and from the results presented here. The latter two studies found that positive and negative images provoke a pupillary contraction to follow by a slow recovery to baseline, with negative images not generating pupillary dilatation whatsoever. Also, those studies found that pupillary contraction showed the same intensity of response for all conditions, reaching a comparable peak for pupillary contraction. Dietz et al. (2011) showed very different curves for each emotional category, with an initial pupil contraction for positive images and initial pupil dilatation for neutral and negative images, followed by a slow recuperation to baseline. Our results are consistent with this study in that different emotional images resulted in a differential contraction response, but differ from all previous studies, in that a steady state response was quickly reached after 2 s following the onset of the emotional images.

There are several methodological issues that can explain, in part, the differences observed between these results. First of all, we deem that a normalization procedure is essential to directly compare pupillary signals between different individuals. Pupillary size can indeed be reported in their actual size for a single individual. Slight variations in the camera’s location will critically result in differences in the actual pupil size. Also, different individuals will exhibit different overall pupil sizes and variable pupil reactivity to the same luminance variation. Another methodological difference relates to the fact that we recorded pupil size at 500 Hz compared to 60 Hz in the other studies. While all these frequencies should properly capture the slow changes occurring during pupillary dilation, we do not have information about how this may impact contraction measurements. On the other hand, the last two studies provided no information about how eye blinks were processed in the data. These eye events evoked fast and strong pupil diameter data changes, which needed to be either removed or corrected. The Dietz et al. (2011) study did remove blinks and other artifacts but presented their data by subtracting the average pupillary response computed over all images, from the average response to the each group of emotional images. The factor that may be considered the most relevant to explain some of the differences between our findings and previous studies is that unlike the other studies, we employed full colored ecological pictures instead of grayscale images, also controlling by luminance distributions among different pictures sets. This can be considered as a major difference in the context of psychological studies of emotional processing.

In work reported here, we found that mindfulness MP exhibited a larger pupillary contraction to negative images, along with a smaller dilatation response. Thus, pupillary responses to negative images were more similar to the response to neutral or positive images. This could be explained by a weaker manifestation of sympathetic nervous system (SNS) activity in this group, indicating that MP subjects showed a decreased noradrenergic reactivity than the group of NM to unpleasant images. This is consistent with other studies, in which the SNS has been associated with emotional arousal, causing greater pupil dilation (Bradley et al., 2008). However, our results showed consistently smaller, but no significant, differences in maximum pupil dilatation between MP and NM subjects. Interestingly, data variability in the meditators group was much lower than the control group, suggesting that autonomic responses in this group could be more homogeneous. Also, these differences in pupillary responses between MP and NM could be explained by the subjective evaluation of emotional images. MP perceive negative images as less activating than NM, which could be reflected in a lower pupillary dilation. The findings of Partala and Surakka (2003) also show an association between the magnitude of pupil response and the amount of emotional arousal felt in participants.

It is not clear whether both components of the autonomic responses are involved in this differential behavior. One possibility is that the meditative practice could be producing an attenuation of SNS activation without affecting activation of the parasympathetic nervous system. The greater pupillary contraction that occurred in our MP subjects when exposed to negative images compared to NM may be due not to a weaker sympathetic response, but also to an earlier and more intense response of the parasympathetic system. Meditation, according to Wallace (1970), is a practice that is accompanied by physiological changes related to oxygen consumption, HR, skin resistance and some EEG frequencies. Tang et al. (2009) noted that the parasympathetic nervous system activity increases during this practice, as it increases the HRV, decreased respiratory rate, breathing amplitude increases and decreases the conductivity of the skin.

One of the effects of meditation practice is the acquisition of emotional regulation strategies. Emotion regulation refers to any process that influences the onset, offset, magnitude, duration, intensity, or quality of one or more aspects of the emotional response (Gross and Barrett, 2011). Here, the time of recovery to baseline levels from emotional stimulation or physical stress is a critical component to talk about emotional regulation. Meditation techniques help the individual to rapidly back to a state of balance (Desbordes et al., 2015), supporting our results showing that MP has a faster physiological recovery of the pupil to baseline levels compared to NM. This stabilization in less time reveals a better adaptive capacity to emotional changes and emotional plasticity.

Unlike the Bradley et al. (2008) study, we did not find any significant changes in HR. These authors showed that negative but not positive images modified HRs compared to neutral images. One possibility for this discrepancy relates to the mismatch between heart frequency and pupillary changes, where the estimation of heart changes are typically prone to large variances, which were not reported in the study above. In our study, we did find that the basal HR of MP subjects was significantly lower than HR of NM subjects. Our results are congruent with the findings of Tang et al. (2009) who reported that after 5 days of training subject on meditative practices, they showed significantly lower HRs, greater belly respiratory amplitudes, and lower chest respiratory rates.

Some limitations of this study and in meditation studies, in general, are the small samples sizes due to the difficulty to find a group of meditators that satisfy the request of the study. Nevertheless, as mentioned before, in this study MP has less variability in their pupillary dynamics than NM. It would be interesting to see if a more restricted characterization of the NM group (e.g., waiting list for a meditation course) affects the homogeneity of pupillary response.

Even so, the results presented here are consistent with previous work (Lutz et al., 2008a, 2009; Desbordes et al., 2015) demonstrating that the continuous and methodical training of meditation as an attentional practice modulates the tone and activity of the autonomic nervous system and emotional responses and recovery. This modulation is reflected in different physiological processes, one of them being the pupil diameter, thus validating the use of Pupillometry as a measure of autonomic changes during emotional processing. Although depth understanding on the mechanism of this modulatory phenomena is still lacking, for example it would be interesting evaluate other autonomic variables like respiration, which could also be an important marker of a meditative state. It is also important to note that these observed changes may not only be utilized as a parameter to measure the effectiveness of meditation, but to evaluate other potential health benefits in the well-being of patients.

## Author Contributions

AV-R, CC and PEM designed the study. AV-R and PEM carried out data acquisition for the study. AV-R and EPB analyzed the data. AV-R and PEM wrote the manuscript.

## Conflict of Interest Statement

The authors declare that the research was conducted in the absence of any commercial or financial relationships that could be construed as a potential conflict of interest.
